# A New Era of Hope for the World's Most Neglected Diseases

**DOI:** 10.1371/journal.pmed.0020323

**Published:** 2005-09-08

**Authors:** The PLoS Medicine Editors

## Abstract

There are good reasons to be optimistic that tropical infectious diseases can be controlled, argue the *PLoS Medicine* editors.

International health agencies have designated a group of 13 tropical infections as neglected diseases (Text S1). These diseases have much in common: they affect the world's poorest people (and are a cause of poverty); they are disabling, disfiguring, and stigmatizing; there is a shortage of safe and effective treatments; and affected patients have represented the lowest-priority markets for Western pharmaceutical manufacturers. Bernard Pécoul of the Drugs for Neglected Diseases Initiative (DNDi) has called these patients “the forgotten people.”

The conventional wisdom is that the outlook for such patients is hopeless. As one of us has argued, “[They] have no purchasing power, no vocal advocacy group is pleading for their needs, and no strategic interests—military or security—are driving concern about these conditions” (BMJ 325: 176–177). Hence, only 13 new drugs have been developed for neglected tropical diseases since 1975, and they have garnered little international attention compared with “the big three” global health threats: HIV/AIDS, tuberculosis, and malaria.

Fortunately, there is good news to report. The “2005 reality,” argues Mary Moran of the London School of Economics in an article in *PLoS Medicine* (DOI:  10.1371/journal.pmed.0020302), is that an explosion of public–private partnerships for health has dramatically altered the landscape of neglected-disease drug development. Her analysis shatters a common illusion—that industry is profoundly disinterested in developing drugs for neglected diseases. “Long-held beliefs on neglected-disease drug development activity are no longer accurate,” writes Moran.

According to her analysis, 63 neglected-disease drug projects were under way at the end of 2004. Three-quarters are being conducted by public–private partnerships that often involve multinational or small-scale commercial firms. Twenty new products are already in clinical trials, including half at the phase III or registration stage. Assuming there is sufficient funding, at standard attrition rates these projects would be expected to deliver eight or nine new neglected-disease drugs within the next five years. “The post-2000 renewal of neglected-disease R&D activity is good news for patients with neglected diseases,” writes Moran.

There are even more reasons for optimism. Firstly, many countries affected by neglected diseases, such as Brazil, Egypt, and India, now have the infrastructure to conduct their own neglected-disease research. Morel and colleagues have called these the “innovative developing countries” (Science 309: 401–404), and say they are now reaping the benefits of decades of investment in education, health research infrastructure, and manufacturing capacity. These countries can begin controlling their endemic tropical diseases themselves by developing their own treatments and vaccines with only modest technical or financial assistance from more developed countries.

Secondly, in addition to the expectation that new tools will be developed to control neglected diseases, there is a surge of interest in maximizing the effectiveness of existing tools. This interest focuses on the idea of taking the disparate vertical control programs, each targeting a specific neglected disease, and delivering them in one integrated package. For example, important work is under way to examine the impact of four drugs—albendazole, praziquantel, azithromycin, and ivermectin—in a single delivery mechanism in order to simultaneously target lymphatic filariasis, onchocerciasis, soil-transmitted helminthiases, schistosomiasis, and trachoma (Lancet 365: 1029–1030).

Thirdly, and perhaps most importantly, the neglected diseases have at long last caught the attention of the international development community. The penny has finally dropped: neglected-disease control will be crucially important to achieving many of the Millenium Development Goals, and investment into neglected diseases must become a priority for donors. On June 8, 2005, DNDi launched an appeal, signed by 17 Nobel laureates among others, calling on donors to create a new fund of $3 billion a year for neglected-disease R&D (http://www.researchappeal.org), while the Commission for Africa urged donors to “ensure that there is adequate funding for the treatment and prevention of parasitic diseases.”

It is therefore encouraging that the Gleneagles Communiqué, arising out of this year's G8 summit, specifically called for increased investment to encourage the development of tools for neglected-disease control. Another sign that donors are taking notice is that the US Senate recently passed a foreign operations appropriations bill that included $30 million for neglected diseases. This successful appropriation was in large part due to the advocacy of the Global Health Council, Eric Otteson at Emory University, and Peter Hotez at George Washington University.

There is one element that will be crucial to the success of all of these new initiatives—the ability to share research results and policy discussions freely across the globe, without access barriers. *PLoS Biology* and *PLoS Medicine* are committed to publishing the highest-quality basic and clinical research on neglected diseases, and PLoS is exploring the feasibility of launching a new journal devoted specifically to these diseases. The “2005 reality” is that there is an unprecedented window of opportunity for neglected-disease control, and PLoS will play its part.

## Supporting Information

Text S1 The 13 Neglected Tropical Diseases (25 KB DOC).

