# Supplementary material for: A New Era of Hope for the World's Most Neglected Diseases
Source: PLoS Med. 2005 Sep 8;2(9):e323. doi: 10.1371/journal.pmed.0020323 (PMC1198044; doi:10.1371/journal.pmed.0020323)
Supplement: Supplementary file 1 [file pmed.0020323.sd001.doc]

**Text S1. The Thirteen Neglected Tropical Diseases**

- Kala-azar (leishmaniasis)
- African Sleeping Sickness (African trypanosomiasis)
- Chagas Disease (American trypanosomiasis)
- Schistosomiasis
- Lymphatic Filariasis (elephantiasis)
- Onchocerciasis (river blindness)
- Drancunculiasis (guinea worm)
- Soil-transmitted Helminthiases: Ascariasis (roundworm), Trichuriasis (whipworm), and hookworm
- Leprosy
- Buruli Ulcer
- Trachoma
